# Inoculation of *Azospirillum brasilense* associated with silicon as a liming source to improve nitrogen fertilization in wheat crops

**DOI:** 10.1038/s41598-020-63095-4

**Published:** 2020-04-09

**Authors:** Fernando Shintate Galindo, Salatiér Buzetti, Willian Lima Rodrigues, Eduardo Henrique Marcandalli Boleta, Vinicius Martins Silva, Renan Francisco Rimoldi Tavanti, Guilherme Carlos Fernandes, Antônio Leonardo Campos Biagini, Poliana Aparecida Leonel Rosa, Marcelo Carvalho Minhoto Teixeira Filho

**Affiliations:** São Paulo State University (UNESP), College of Engineering of Ilha Solteira, Department of Plant Health, Rural Engineering, and Soils, P.O. BOX 15385-000, Av. Brasil Sul, 830 - Centro, Ilha Solteira, state of São Paulo Brazil

**Keywords:** Field trials, Leaf development, Environmental impact

## Abstract

This research was developed to investigate whether inoculation with *Azospirillum brasilense* in combination with silicon (Si) can enhance N use efficiency (NUE) in wheat and to evaluate and correlate nutritional and productive components and wheat grain yield. The study was carried out on a Rhodic Hapludox under a no-till system with a completely randomized block design with four replications in a 2 × 2 × 5 factorial scheme: two liming sources (with Ca and Mg silicate as the Si source and limestone); two inoculations (control - without inoculation and seed inoculation with *A*. *brasilense*) and five side-dress N rates (0, 50, 100, 150 and 200 kg ha^−1^). The results of this study showed positive improvements in wheat growth production parameters, NUE and grain yield as a function of inoculation associated with N rates. Inoculation can complement and optimize N fertilization, even with high N application rates. The potential benefits of Si use were less evident; however, the use of Si can favour N absorption, even when associated with *A*. *brasilense*. Therefore, studies conducted under tropical conditions with Ca and Mg silicate are necessary to better understand the role of Si applied alone or in combination with growth-promoting bacteria such as *A*. *brasilense*.

## Introduction

Wheat (*Triticum aestivum* L.) is a grain crop that has significant economic importance among cool season cereals due to its high grain-yield capacity^1,2^. In addition to maize (*Zea mays* L.) and rice (*Oryza sativa* L.), wheat is responsible for above 50% of human calories and is an important food source in regions with rapid population growth, such as Africa, Asia and Latin America^3^. However, in the last five years, 51.2% of Brazil’s wheat consumption was imported from Argentina, the United States, Russia and Paraguay^4^. Similar to other grain crops, wheat production under savanna conditions is limited by nitrogen (N) availability^5^. In addition, it has been estimated that as much as 17% of the total operating profit of the wheat being produced on the Brazilian savanna is due to side-dress N application^6^. The production and over application of N fertilizer contribute to a series of environmental problems, such as air pollution, greenhouse gas emissions, global warming, contamination of water bodies and eutrophication^7–9^. Therefore, integrated N management strategies could contribute significantly to improving N use efficiency (NUE) under savanna conditions, particularly for wheat, which has high N requirements^10,11^.

Bacteria with multiple plant-growth promoting traits (PGPB, plant-growth promoting bacteria) can improve NUE and increase the growth and grain yields of cereal crops under tropical conditions^1,12–14^. Numerous PGPB genera show positive relations with different plant species (for example, *Azospirillum*, *Rhizobium*, *Bradyrhizobium*, *Bacillus* and *Pseudomonas*)^15^. The genus *Azospirillum* is currently one of the most commercially employed PGPB^16,17^. This microorganism can colonize several plant species and significantly improves their growth, development and yield under field conditions^16,18^. Previous studies with *Azospirillum* highlight its capacity to fix N_2_, as well as its benefits in terms of promoting plant growth via the synthesis of phytohormones, such as auxins, gibberellins, cytokinins, ethylene and salicylic acid, and nutrient availability increases^2,9,17,19,20^. Additionally, *Azospirillum* sp. has been related with reduced biotic and abiotic stresses, for example, increasing plant resistance to phytopathogens, salinity and drought and reducing the oxidative damage caused by an increase in reactive oxygen species^17,19,21,22^. Munareto *et al*.^23^ verified the positive influence of N side-dress fertilization combined with *Azospirillum brasilense* seed inoculation in wheat and reported an average yield increase of 36.8% and 13.5% when the inoculation was associated with N application rates of 70 and 140 kg ha^−1^, respectively. Additionally, Galindo *et al*.^24^ reported an average NUE increase of 51.2% when the inoculation with *A*. *brasilense* was associated with N application rates varying between 50 and 200 kg ha^−1^.

In recent years, the use of silicon (Si) fertilizers has been reported to improve plant growth and development with greater N uptake and NUE in cereal crops^25–28^. Silicon application has been associated with increased plant resistance to abiotic and biotic stresses, such as attacks from insects and pathogens, salinity, drought, flooding, and high and low temperatures^28–31^. Si enhances primary metabolism by decreasing the transpiration rate^32^ and improving photosynthesis^31,33^ and nutrient uptake^34^ as well as secondary metabolism by stimulating the production of phenolic compounds with either antioxidant (for example, flavonoids) or structural (for example, lignin and cellulose) functions^31,35,36^. Silicon has also been reported to stimulate germination^28,37^ and increase root length^28,37^, the number of leaves and plant architecture^36,38^. Calcium and magnesium silicate as a liming source can correct soil acidity; increase soil pH, extractable levels of Si, Ca, Mg and P; and decrease the harmful effects of toxic heavy metals such as Al, Fe and Cd^39–42^.

Silicon is the second most abundant element in the earth’s crust after oxygen^25,43^. However, Si availability in tropical soils is limited mainly by the high levels of weathering and desilication of primary silicate minerals^44–46^. Additionally, a large turnover of Si occurs in terrestrial and semi aquatic ecosystems, with plants and plant litter decomposition being the driving force^25^. Therefore, studies on Si applications aiming to increase Si replacement, NUE and N uptake under tropical conditions should be performed. Recently, research on the interaction of Si application and PGPB inoculation has increased^47–50^. Etesami^47^ suggests that the combined use of Si and PGPB can be a powerful and sustainable strategy to enhance plant growth under sub-optimal conditions. Rezakhani *et al*.^49,50^ concluded that *Pseudomonas* sp. associated with Si application can improve wheat and sorghum (*Sorghum bicolor* (L.) Moench) development. Galindo *et al*.^2^ studied inoculation methods associated with Ca and Mg silicate and reported an average wheat grain yield increase of 6.7% when seed inoculation and silicate application were performed. Therefore, new studies with Si applications associated with inoculation need to be performed to improve NUE, wheat development and grain yield. Additionally, new studies are needed to define how much N fertilizer needs to be applied when Si is applied in association with A. *brasilense* for optimum wheat grain yields. The hypothesis of this study was that the application of Si in combination with *A. brasilense* inoculation would improve NUE and reduce the amount of N required for maximum wheat production under savanna conditions.

## Results

### Nutritional evaluations

**Figure 1:**
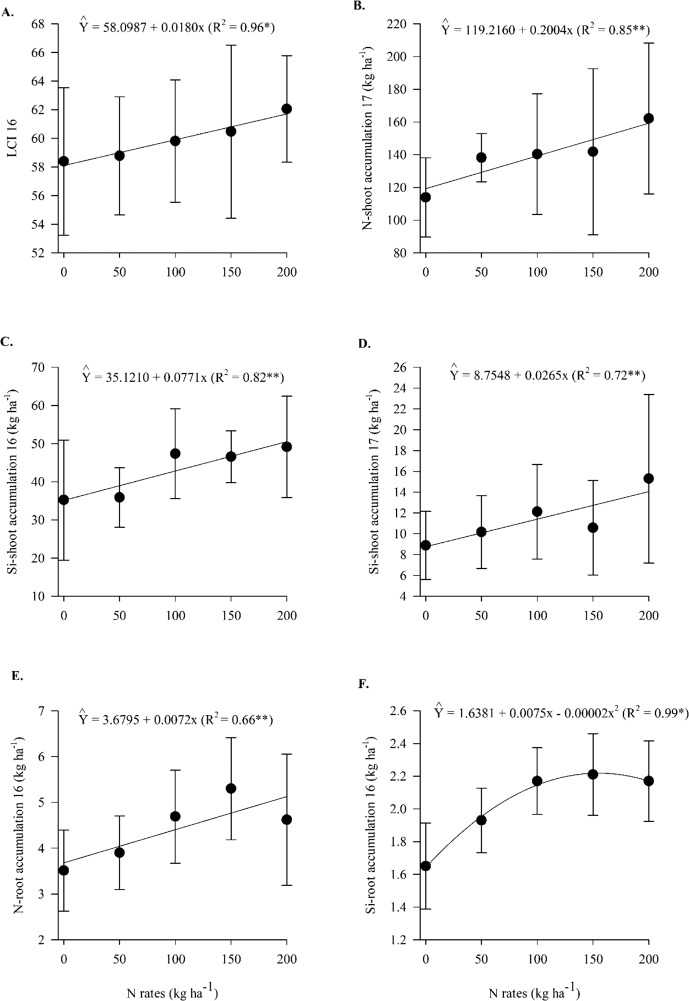
Leaf chlorophyll index (LCI) in 2016 (**A**), N-shoot accumulation in 2017 (**B**), Si-shoot accumulation in 2016 (**C**) and 2017 (**D**), N-root accumulation in 2016 (**E**) and Si-root accumulation in 2016 (**F**) as a function of N rates. Error bars indicate the standard deviation of the mean (n = 4).

**Figure 2:**
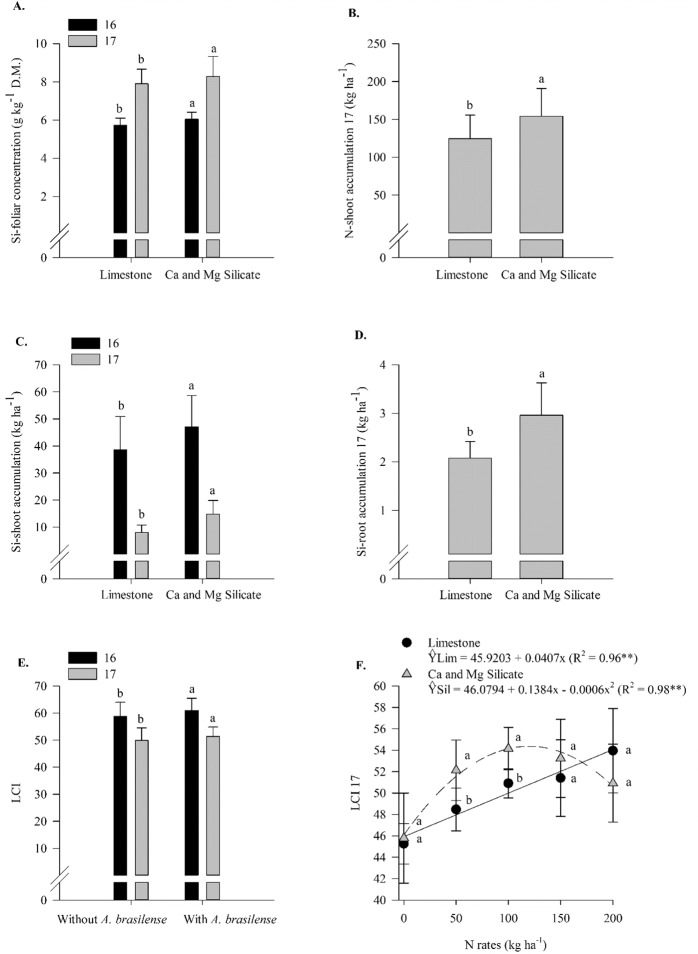
Si-foliar concentration in 2016 and 2017 (**A**), N-shoot accumulation in 2017 (**B**), Si-shoot accumulation in 2016 and 2017 (**C**), Si-root accumulation in 2017 (**D**) as a function of liming sources, LCI in 2016 and 2017 (**E**) as a function of inoculation with *A*. *brasilense* and interaction between N rates and liming sources in LCI in 2017 (**F**). The letters correspond to a significant difference at 5% probability level (*p* ≤ 0.05). ** and *: significant at *p* < 0.01 and *p* < 0.05, res*p*ectively. Error bars indicate the standard deviation of the mean (*n* = 4). L.S.D. (least significant difference) = 0.16 and 0.24 (**A**), 17.13 (**B**), 6.05 and 2.20 (**C**), 0.24 (**D**), 1.96 and 1.44 (**E**) and 3.21 (**F**).

The leaf chlorophyll index and N-root accumulation in 2016, N-shoot accumulation in 2017 and Si-shoot accumulation in both years responded linearly to N rates (Supplementary Table 1, Fig. 1A–E). Silicon accumulation in roots in 2016 responded non-linearly to N rates (up to 187 kg N ha^−1^) (Supplementary Table 1, Fig. 1F). In comparison to limestone application, silicate application resulted in greater Si-foliar concentration and Si-shoot accumulation in both years and greater N-shoot and Si-root accumulation in 2017 (Supplementary Table 1, Fig. 2A–D). In comparison to the no inoculation treatments, the inoculation treatments with *A*. *brasilense* provided greater (Leaf chlorophyll index) LCI in both years (Supplementary Table 1, Fig. 2E).

**Figure 3:**
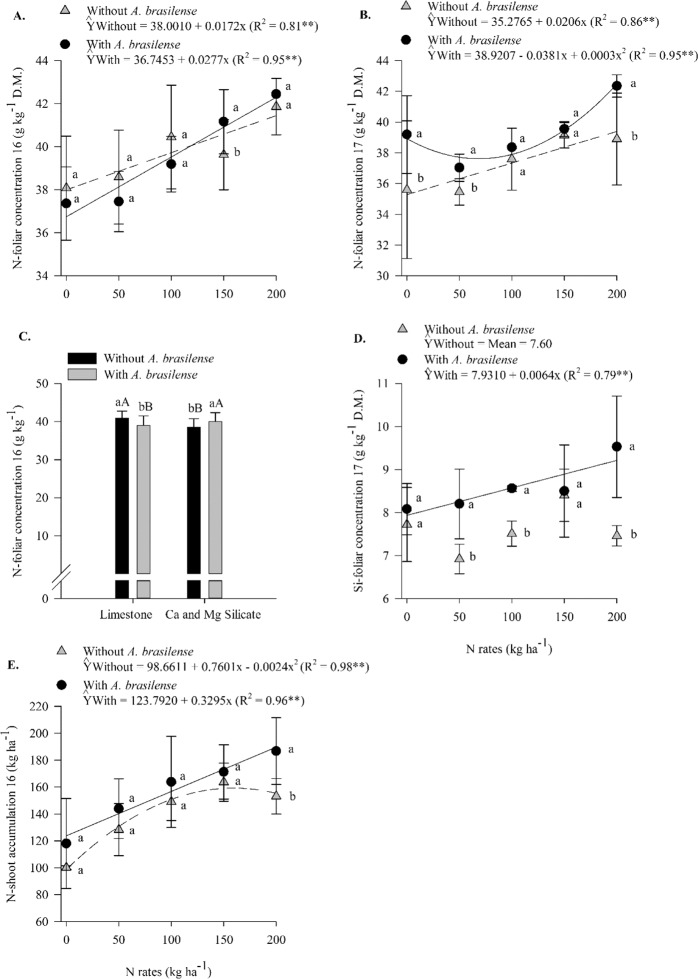
Interaction between N rates and inoculation with *A*. *brasilense* in N-foliar concentration in 2016 (**A**) and 2017 (**B**), interaction between liming sources and inoculation in N-foliar concentration in 2016 (**C**), interaction between N rates and inoculation in Si-foliar concentration in 2017 (**D**) and interaction between N rates and inoculation in N-shoot accumulation in 2016 (**E**). The letters correspond to a significant difference at 5% probability level (*p* ≤ 0.05). Uppercase letters indicate difference between inoculations, and lowercase letters indicate differences between liming sources, respectively. ** and *: significant at *p* < 0.01 and *p* < 0.05, res*p*ectively. Error bars indicate the standard deviation of the mean (*n* = 4). L.S.D. (least significant difference) = 1.53 (**A**), 1.39 (**B**), 0.97 (**C**), 0.24 (**D**) and 26.48 (**E**).

In 2017, silicate application showed greater LCI when associated with 50 and 100 kg N ha^−1^ (Supplementary Table 1, Fig. 2F). The leaf chlorophyll index responded linearly to N rates when dolomitic limestone was applied and non-linearly when Ca and Mg silicate were applied (up to 115 kg N ha^−1^) (Supplementary Table 1, Fig. 2F). In 2016, the inoculation with *A*. *brasilense* associated with 150 kg N ha^−1^ resulted in a greater N-foliar concentration compared to that in the no inoculation treatments (Supplementary Table 1, Fig. 3A). Nitrogen foliar concentrations responded linearly to N rates with and without inoculation (Supplementary Table 1, Fig. 3A). In 2017, in comparison with the no inoculation treatments, the inoculation treatments with *A*. *brasilense* associated with 50 and 200 kg N ha^−1^ resulted in a greater N-foliar concentration (Supplementary Table 1, Fig. 3B). Nitrogen foliar concentration responded linearly to N rates without inoculation and non-linearly with inoculation (Supplementary Table 1, Fig. 3B). In addition, in 2016, in comparison to the silicate application, the limestone application increased the N-foliar concentration compared in the absence of inoculation; however, in comparison to the limestone application, the silicate application increased the N-foliar concentration when inoculation was performed (Supplementary Table 1, Fig. 3C). In 2017, in comparison to the no inoculation treatments, the inoculation treatments with *A*. *brasilense* associated with 50, 100, and 200 kg N ha^−1^ resulted in greater Si-foliar concentrations (Supplementary Table 1, Fig. 3D). Silicate foliar concentration responded linearly to N rates when inoculation was performed (Supplementary Table 1, Fig. 3D). In 2016, inoculation associated with 200 kg N ha^−1^ increased N-shoot accumulation compared to that in the no inoculation treatments (Supplementary Table 1, Fig. 3E). Nitrogen accumulation in shoots responded linearly to N rates when the treatments were inoculated and responded non-linearly when the treatments were not inoculated (up to 158 kg N ha^−1^) (Fig. 3E).

**Figure 4:**
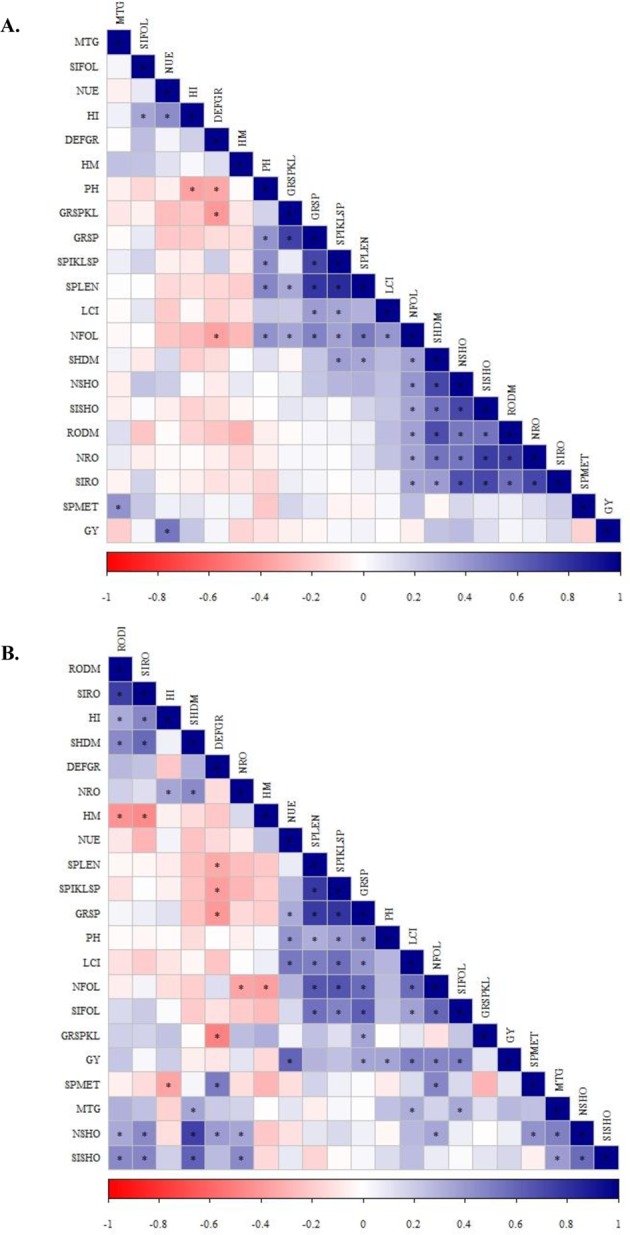
Heatmap showing the Pearson correlation among nutritional evaluations, productive components, nitrogen use efficiency and wheat grain yield for non-inoculated treatments in 2016 (**A**) and in 2017 (**B**). LCI = leaf chlorophyll index, NFOL = N-leaf concentration, SIFOL = Si-leaf concentration, NSHO = N-shoot accumulation, SISHO = Si-shoot accumulation, NRO = N-root accumulation, SIRO = Si-root accumulation, SHDM = shoot dry matter, RODM = root dry matter, PH = plant height, SPMET = spikes per meter, SPLEN = spike length, DEFGR = defective grains, SPIKLSP = spikelets per spike, GRSPIKL = grains per spikelet, GRSP = grains per spike, HM = hectoliter mass, MTG = mass of 1000 grains, HI = Harvest index, NUE = Nitrogen use efficiency and GY = grain yield.

In 2016, Pearson’s linear correlation was positive between the N-foliar concentration and plant height, N-foliar concentration and grains per spikelet, N-foliar concentration and shoot dry matter, N-foliar concentration and root-dry matter, N-foliar concentration and Si-shoot concentration, N-foliar concentration and N-root accumulation, N-foliar concentration and Si-root accumulation, N-root accumulation and root dry matter, N-root accumulation and Si-root accumulation, Si-foliar concentration and harvest index and negative between defective grains and N-foliar concentration for the no inoculation treatments (Fig. 4A). In 2017, Pearson’s linear correlation was positive between LCI and NUE, LCI and spike length, LCI and Si-foliar concentration, LCI and grain yield, LCI and mass of 1000 grains, Si-foliar concentration and spike length, Si-foliar concentration and spikelets per spike, Si-foliar concentration and grains per spike, Si-foliar concentration and grain yield, Si-foliar concentration and mass of 1000 grains, N-shoot accumulation and defective grains, N-shoot accumulation and spikes per meter, N-shoot accumulation and mass of 1000 grains, N-foliar concentration and Si-foliar concentration, N-foliar concentration and grain yield, N-foliar concentration and spikes per meter, Si-shoot accumulation and mass of 1000 grains, Si-root accumulation and harvest index, and N-root accumulation and harvest index and was negative between N-foliar concentration and N-root accumulation, N-foliar concentration and hectoliter mass, and Si-root accumulation and harvest index for non-inoculated treatments (Fig. 4B). In both seasons (2016 and 2017), Pearson’s linear correlation was positive between LCI and grains per spike, LCI and spikelets per spike, LCI and N-foliar concentration, N-foliar concentration and grains per spike, N-foliar concentration and spikelets per spike, N-foliar concentration and spike length, N-foliar concentration and N-shoot accumulation, N-shoot accumulation and shoot dry matter, N-shoot accumulation and root dry matter, N-shoot accumulation and Si-shoot accumulation, N-shoot accumulation and N-root accumulation, N-shoot accumulation and Si-root accumulation, Si-shoot accumulation and shoot dry matter, Si-shoot accumulation and root dry matter, Si-shoot accumulation and N-root accumulation, Si-shoot accumulation and Si-root accumulation, Si-root accumulation and shoot dry matter, Si-root accumulation and root dry matter, and N-root accumulation and shoot dry matter for non-inoculated treatments (Fig. 4A,B).

**Figure 5:**
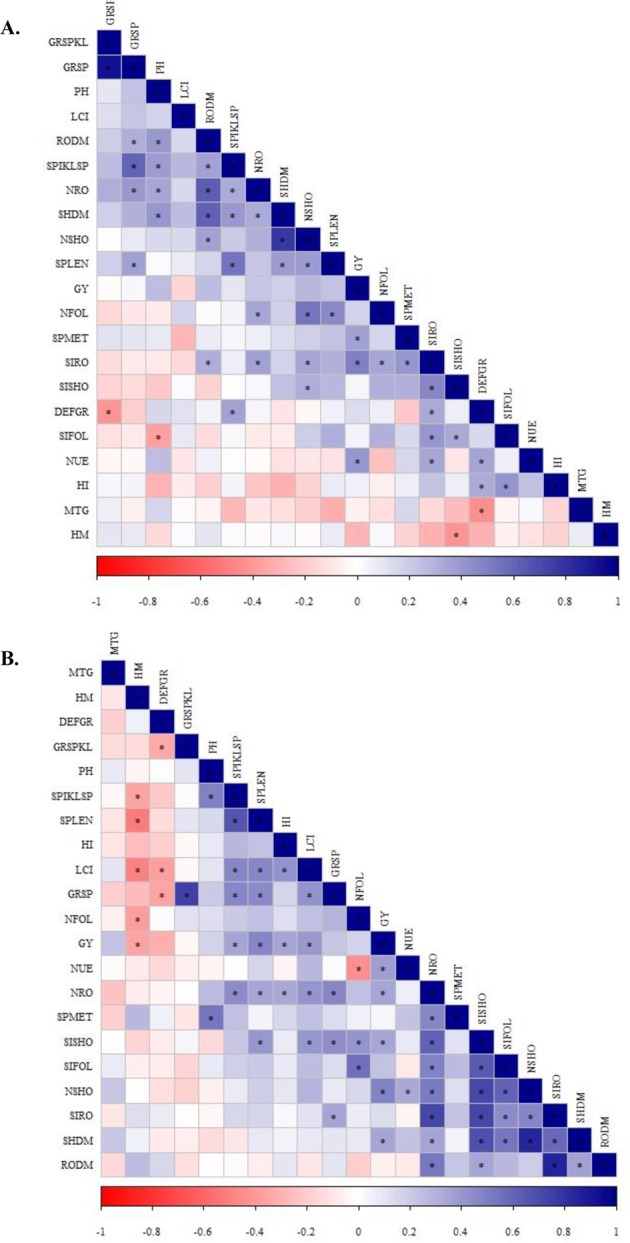
Heatmap showing the Pearson correlation among nutritional evaluations, productive components, nitrogen use efficiency and wheat grain yield for *A*. *brasilense* inoculated treatments in 2016 (**A**) and in 2017 (**B**). LCI = leaf chlorophyll index, NFOL = N-leaf concentration, SIFOL = Si-leaf concentration, NSHO = N-shoot accumulation, SISHO = Si-shoot accumulation, NRO = N-root accumulation, SIRO = Si-root accumulation, SHDM = shoot dry matter, RODM = root dry matter, PH = plant height, SPMET = spikes per meter, SPLEN = spike length, DEFGR = defective grains, SPIKLSP = spikelets per spike, GRSPIKL = grains per spikelet, GRSP = grains per spike, HM = hectoliter mass, MTG = mass of 1000 grains, HI = Harvest index, NUE = Nitrogen use efficiency and GY = grain yield.

For the inoculated treatments, in 2016, Pearson’s linear correlation was positive between Si-root accumulation and N-foliar concentration, Si-root accumulation and spikes per meter, Si-root accumulation and defective grains, Si-root accumulation and NUE, Si-root accumulation and grain yield, N-shoot accumulation and root dry matter, N-shoot accumulation and spike length, N-shoot accumulation and N-foliar concentration, N-foliar concentration and spike length, N-foliar concentration and N-root accumulation, N-root accumulation and plant height, and Si-foliar concentration and harvest index and was negative between Si-foliar concentration and plant height and S-shoot accumulation and hectoliter mass (Fig. 5A). In 2017, Pearson’s linear correlation was positive between LCI and spikelets per spike, LCI and spike length, LCI and harvest index, LCI and grains per spike, LCI and grain yield, LCI and N-root accumulation, LCI and Si-shoot accumulation, N-root accumulation and spike length, N-root accumulation and harvest index, N-root accumulation and grain yield, N-root accumulation and spikes per meter, N-root accumulation and Si-shoot accumulation, N-root accumulation and Si-foliar concentration, N-root accumulation and N-shoot accumulation, Si-shoot accumulation and spike length, Si-shoot accumulation and grains per spike, Si-shoot accumulation and N-foliar concentration, Si-shoot accumulation and grain yield, Si-shoot accumulation and shoot dry matter, Si-shoot accumulation and root dry matter, Si-foliar concentration and N-foliar concentration, Si-foliar concentration and N-shoot accumulation, Si-foliar concentration and shoot dry matter, N-shoot accumulation and grain yield, N-shoot accumulation and NUE, and Si-root accumulation and grains per spike and negative between LCI and hectoliter mass, LCI and defective grains, N-foliar concentration and hectoliter mass, and N-foliar concentration and NUE for inoculated treatments with *A*. *brasilense* (Fig. 5B). In both crop seasons (2016 and 2017), Pearson’s linear correlation was positive between N-root accumulation and grains per spike, N-root accumulation and root dry matter, N-root accumulation and spikelets per spike, N-root accumulation and shoot dry matter, N-root accumulation and Si-root accumulation, N-shoot accumulation and shoot dry matter, N-shoot accumulation and Si-root accumulation, N-shoot accumulation and Si-shoot accumulation, Si-root accumulation and root dry matter, Si-root accumulation and Si-shoot accumulation, Si-root accumulation and Si-foliar concentration and Si-shoot accumulation and Si-foliar concentration for the inoculated treatments (Fig. 5A.B).

### Productive components

**Figure 6:**
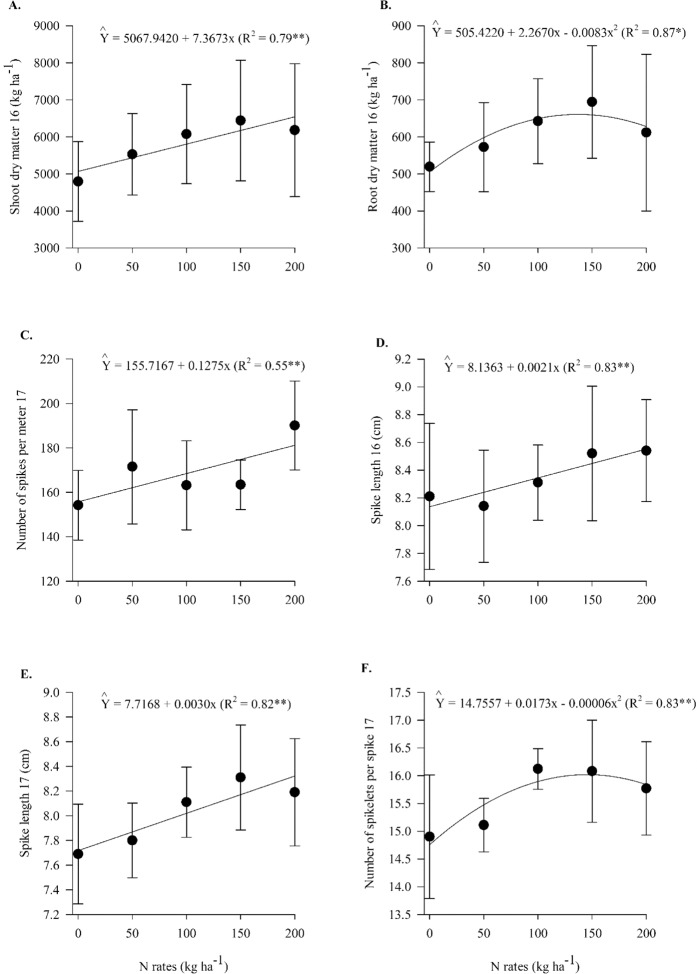
Shoot dry matter in 2016 (**A**), root dry matter in 2016 (**B**), number of spikes per meter in 2017 (**C**), spike length in 2016 (**D**) and 2017 (**E**) and number of spikelets per spike in 2017 (**F**) as a function of N rates. Error bars indicate the standard deviation of the mean (*n* = 4).

**Figure 7:**
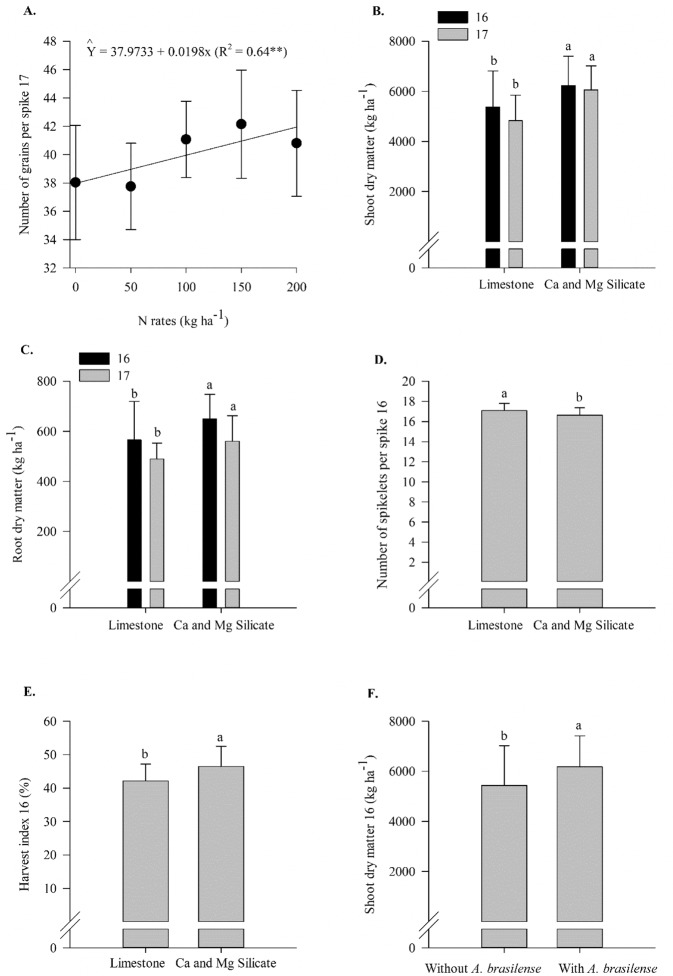
Number of grains per spike in 2017 (**A**) as a function of N rates, shoot dry matter in 2016 and 2017 (**B**), root dry matter in 2016 and 2017 (**C**), number of spikelets per spike in 2016 (**D**), harvest index in 2016 (**E**) as a function of liming sources, and shoot dry matter in 2016 (**F**) as a function of inoculation with *A*. *brasilense*. The letters correspond to a significant difference at 5% probability level (*p* ≤ 0.05). Error bars indicate the standard deviation of the mean (*n* = 4). L.S.D. (least significant difference) = 705.95 and 594.66 (**B**), 70.59 and 39.06 (**C**), 0.40 (**D**), 4.17 (**E**) and 705.95 (**F**).

**Figure 8:**
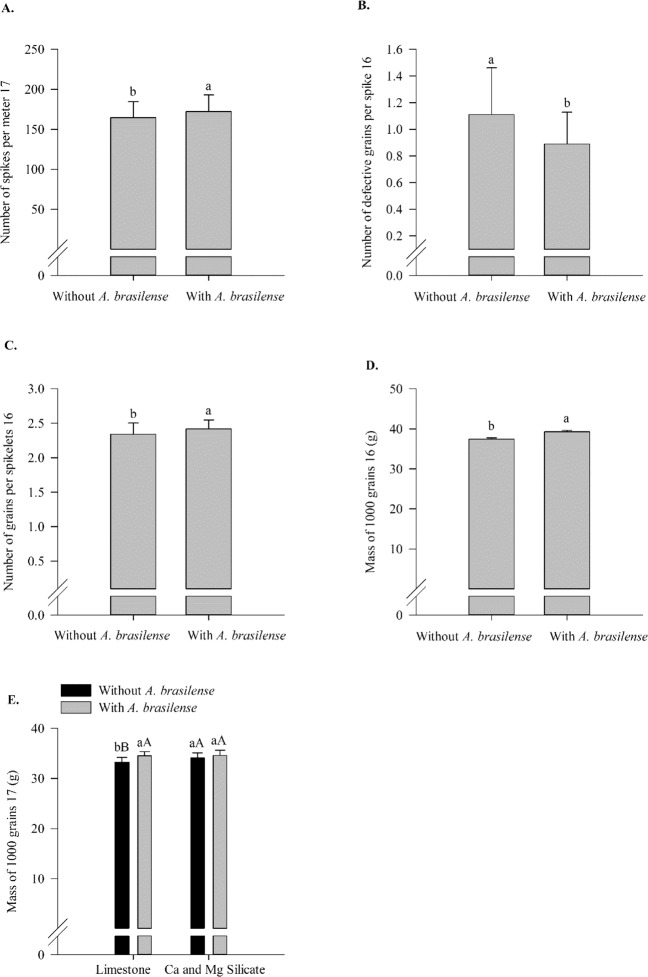
Number of spikes per meter in 2017 (**A**), number of defective grains per spike in 2016 (**B**), number of grains per spikelet in 2016 (**C**), mass of 1000 grains in 2016 (d) as a function of inoculation with *A*. *brasilense*, and interaction between liming sources and inoculation in mass of 1000 grains in 2017 (**E**). The letters correspond to a significant difference at 5% probability level (*p* ≤ 0.05). Uppercase letters indicate difference between inoculations, and lowercase letters indicate differences between liming sources, respectively. Error bars indicate the standard deviation of the mean (*n* = 4). L.S.D. (least significant difference) = 7.45 (**A**) 0.15 (**B**), 0.07 (**C**), 1.50 (**D**) and 0.69 (**E**).

Shoot dry matter in 2016, number of spikes per meter and grains per spike in 2017 and spike length in both years responded linearly to N rates. Root dry matter in 2016 and the number of spikelets per spike responded non-linearly to N rates (up to 137 and 144 kg N ha^−1^, respectively) (Supplementary Table 1, Fig. 6A–F,A). In comparison to the limestone application, the silicate application provided greater shoot and root dry matter in both years and harvest index in 2016; however, limestone showed greater number of spikelets per spike in 2016 (Supplementary Table 1, Fig. 7B–E). In comparison to the no inoculation treatment, the inoculation treatment with *A*. *brasilense* resulted in greater shoot dry matter, grains per spikelet and mass of 1000 grains in 2016 and a greater number of spikes per meter in 2017. In addition, the inoculation reduced the number of defective grains in 2016 (Supplementary Table 1, Fig. 8A–D). In 2017, the silicate application resulted in a greater mass of 1000 grains compared to that with the limestone application in the absence of inoculation (Supplementary Table 1, Fig. 8E). In contrast, limestone application resulted in a greater mass of 1000 grains compared to that with the silicate application when inoculation was performed (Supplementary Table 1, Fig. 8E).

In 2016, Pearson’s linear correlation was positive between spike length and grains per spikelet, spike length and shoot dry matter, shoot dry matter and root dry matter, shoot dry matter and spikelets per spike, spikes per meter and mass of 1000 grains, and harvest index and NUE and was negative between plant height and harvest index and plant height and defective grains for the non-inoculated treatments (Fig. 4A). In 2017, Pearson’s linear correlation was positive between root dry matter and harvest index, root dry matter and shoot dry matter, shoot dry matter and mass of 1000 grains, spikes per meter and defective grains, grains per spike and NUE, grains per spike and grain yield, plant height and NUE and plant height, and grain yield and was negative between root dry matter and hectoliter mass, harvest index and spikes per meter, defective grains and spike length, defective grains and spikelets per spike, and defective grains and grains per spike for the non-inoculated treatments (Fig. 4B). In addition, in both seasons (2016 and 2017), Pearson’s linear correlation was positive between plant height and grains per spike, plant height and spikelets per spike, plant height and spike length, grains per spike and grains per spikelet, grains per spike and spikelets per spike, grains per spike and spike length, and spikelets per spike and spike length and was negative between defective grains and grains per spikelet in both crops for the non-inoculated treatments (Fig. 4A,B).

For the inoculated treatments, in 2016, Pearson’s linear correlation was positive between root dry matter and grains per spike, root dry matter and plant height, root dry matter and spikelets per spike, plant height and shoot dry matter, spikelets per spike and shoot dry matter, spikelets per spike and defective grains, shoot dry matter and spike length, spikes per meter and grain yield, defective grains and NUE, and defective grains and harvest index and was negative between defective grains and mass of 1000 grains (Fig. 5A). In 2017, Pearson’s linear correlation was positive between spikes per meter and plant height, spikelets per spike and grain yield, spike length and grain yield, harvest index and grain yield, and shoot dry matter and grain yield and was negative between hectoliter mass and spikelets per spike, hectoliter mass and spike length, hectoliter mass and grain yield, and defective grains and grains per spike for the inoculated treatments with *A*. *brasilense* (Fig. 5B). Additionally, in both seasons (2016 and 2017), Pearson’s linear correlation was positive between grains per spike and grains per spikelet, grains per spike and spikelets per spike, grains per spike and spike length, spikelets per spike and plant height, spikelets per spike and spike length, and root and shoot dry matter and negative between defective grains and grains per spikelet for inoculated treatments (Fig. 5A,B).

### Nitrogen use efficiency and wheat grain yield

**Figure 9:**
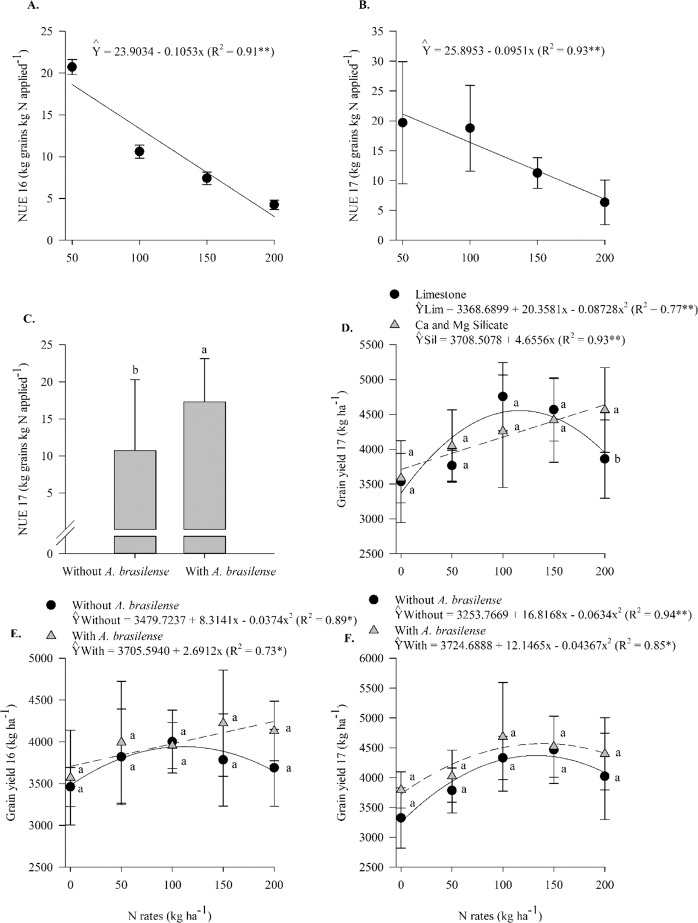
Nitrogen use efficiency (NUE) in 2016 (**A**) and 2017 (**B**) as a function of N rates, NUE in 2017 (**C**) as a function of inoculation with *A*. *brasilense*, interaction between N rates and liming sources in wheat grain yield in 2017 (**D**), interaction between N rates and inoculation in wheat grain yield in 2016 (**E**) and 2017 (**F**). The letters correspond to a significant difference at 5% probability level (*p* ≤ 0.05). ** and *: significant at *p* < 0.01 and *p* < 0.05, res*p*ectively. Error bars indicate the standard deviation of the mean (*n* = 4). L.S.D. (least significant difference) = 2.50 (**C**), 544.65 (**D**), 480.07 (**E**) and 544.65 (**F**).

In both 2016 and 2017, NUE was found to decrease linearly with N rates (Supplementary Table 1, Fig. 9A,B). In 2017, in comparison to the no inoculation treatments, the inoculation treatments resulted in greater NUE, at an average increase of 6.6 kg grains kg N applied^−1^, equivalent to 61.6% (Supplementary Table 1, Fig. 9C). In 2017, in comparison to the limestone application, the silicate application associated with 200 kg N ha^−1^ resulted in greater grain yield(Supplementary Table 1, Fig. 9D). Grain yield responded linearly to N rates when silicate was applied and non-linearly when limestone was applied (up to 117 kg N ha^−1^) (Supplementary Table 1, Fig. 9D). In 2016, grain yield responded linearly to N rates (up to 111 kg N ha^−1^)when inoculation was performed and non-linearly when inoculation was not performed (Supplementary Table 1, Fig. 9E). In 2017, grain yield responded non-linearly to N rates both with inoculation (up to 139 kg N ha^−1^) and without inoculation (up to 134 kg N ha^−1^) (Supplementary Table 1, Fig. 9F). Pearson’s linear correlation was positive between NUE and grain yield in both seasons for the non-inoculated and inoculated treatments with *A*. *brasilense* (Figs. 4A,B and 5A,B).

## Discussion

Applied nitrogen was absorbed as verified by the increased N-leaf concentration, N root and shoot accumulation and LCI and the favoured Si uptake, with an increase in Si-leaf concentration and shoot and root accumulation. In addition, the applied N rates improved wheat development, reflecting a relatively high grain yield as verified by the increased shoot and root dry matter, number of spikes per meter, spike length, spikelets per spike and grains per spike. Nitrogen is the nutrient that is most demanded by wheat plants and directly affects crop development and yield, providing building blocks for the synthesis biomolecules, such as proteins, nucleic acids and chlorophyll^51^. The higher N availability with N application than without N application likely favoured root system development, leading to improved shoot development and wheat grain yield. Increased root dry matter can positively influence root scavenging, which is important for the interception of nutrients in crop systems. The positive Pearson’s correlations obtained in the present study between the N-foliar concentration and accumulations in the shoot and root with the productive components and grain yield support this hypothesis. Nitrogen fertilization has been reported to benefit different cereal crops, such as wheat^5,52^, maize^53,54^, rice^55,56^, barley^57,58^ and sorghum^59,60^. However, the application of N as a side dress must follow best management practices developed for each region. The application of excessive N rates may result in increased N losses due to low utilization by cereal crops^12^, as verified by decreased NUE with increasing N rates.

The specific roles underlying the *A*. *brasilense* effect on N uptake by wheat were not evaluated in the present study; however, it is possible that the higher NUE observed in inoculated plants than in non-inoculated plants was due to its ability to promote plant growth^12,14,61,62^. The *A*. *brasilense* strains Ab-V5 and Ab-V6 carry similar *fix* and *nif* genes that are responsible for fixing N_2_^63^, and both strains share similar genes related to the synthesis of auxins^62,64^. Recently, Pii *et al*.^65^ concluded that the maize inoculation with *A*. *brasilense* provided greater root development, regardless of nitrate concentration in soil solution. In addition, Pii *et al*.^65^ reported that *A*. *brasilense* inoculation counteracted the nitrate uptake inducing phenomenon without affect the overall N uptake to the plant, most likely through supplementation by bacteria-derived ammonium. Therefore, these specific mechanisms could have benefited the ability of wheat plants to more efficiently explore the soil volume and consequently improve nutrient uptake, as verified in some studies using *A*. *brasilense*^14,17,64,66–68^. In the first season, our data showed that grain yield in the inoculated plots at the higher applied N rates (150 and 200 kg N ha^−1^) tended to be greater than that in the non-inoculated plots (11.6 and 12.0%, respectively). The plots that were inoculated showed a slower rate of response to applied N compared with plots that were not inoculated. In the second season, a different behaviour was observed, and a residual effect was likely observed. Considering the isolated effect of inoculation, the increase in wheat grain yield was approximately 225 and 299 kg ha^−1^ in both crops, equivalent to an increase in yield of 6 and 7.5%, respectively. Similar results were reported when the side-dress N application was found to improve wheat yield between 3.1 and 25.2% in *A*. *brasilense* inoculated plants compared with non-inoculated plants^1,23,69–71^. Additionally, inoculation increased NUE in 2017 (an increase of 61.7%) and favoured NUE in 2016 (an increase of 14.8%), reflecting greater grain filling and wheat grain yield, as verified by the increased number of grains per spikelet in 2016, the mass of 1000 grains in 2016 and the reduction in the number of defective grains in 2016. The positive Pearson’s correlation between N-foliar concentration, N-shoot and root accumulation, Si-foliar concentration, and Si-shoot and root accumulation with the productive components when treatments were inoculated with *A*. *brasilense* support this hypothesis. Therefore, our results show that *A*. *brasilense* seed inoculation may be a potential strategy to help improve NUE.

Calcium and Mg silicate were effective in making Si available for wheat plants, as verified by the increased Si-leaf concentration and Si-shoot and root accumulation and favoured N absorption, with an increase in N-shoot accumulation. The positive Pearson’s correlations between the N-foliar concentration and shoot and root accumulation with Si-foliar concentration and accumulation in shoots and roots support this hypothesis. Silicon fertilization can benefit the foliar architecture of plants by improving erectness of leaves, leading to a greater light interception, reducing self-shading and lodging, postponing senescence and improving photosynthesis^72–74^. Our results showed that most of the Si that was absorbed by the plant accumulated in the leaf tissue. The accumulated Si is deposited within the leaf epidermis, following the plant transpiration flux. Inside plant epidermis, Si becomes condensed into a polymerized silica gel (SiO_2_ *n*H_2_O) known as a phytolith that is immobile and makes up a protective structural layer in plant cell walls^45,75,76^. Some plant species, such as wheat, can take up and translocate large quantities of Si in the aboveground tissue due to specific Si transporters^77^. Shoot Si uptake for wheat may vary between 30 and 133 kg Si ha^−1^ depending on the Si source applied^2,43,45,78^. The N/Si ratio in the leaf tissue, shoot and root was, on average, in both seasons, 5.5, 5.3 and 2.3, respectively. The N-leaf concentration was greater than the Si-leaf concentration (39.62 and 38.32 for N and 5.88 and 8.09 g kg^−1^ of dry mass [D.M.] for Si in 2016 and 2017, respectively). Howeverm the Si concentration in foliar tissue was near the appropriate concentration range for Ca (2.5–10 g kg^−1^ D.M.) and above the appropriate concentration for Mg (1.5–4.0 g kg^−1^ of D.M.), which are the third and fourth nutrients, respectively, most absorbed by wheat^79^.

The greater Si uptake associated with increased N absorption and LCI due to the silicate application positively influenced the shoot and root dry matter and harvest index. In comparison to the limestone application, the silicate application associated with 200 kg N ha^−1^ also resulted in a greater grain yield (an average increase of 18.2%). In addition, the silicate application did not negatively affect the inoculation with *A*. *brasilense* and showed greater N-leaf concentration in 2016 when inoculation was performed than when inoculation was not performed. Recently, the interaction of Si application and PGPB inoculation have been determined to be a sustainable strategy to enhance plant growth under sub-optimal conditions^47^. Mahmood *et al*.^80^ verified that the combined inoculation of *Bacillus drentensis* with 2 kg Si ha^−1^ led to substantial intensification of mung bean (*Vigna radiata* (L.) Wilczek) growth, physiology, and yield under salinity-affected conditions. Galindo *et al*.^2^ studied inoculation methods associated with Si application and reported an average wheat grain yield increase of 6.7% when seed inoculation and Si application were performed. Additionally, calcium and magnesium silicate as a liming source can correct soil acidity; increase soil pH, extractable levels of Si, Ca, Mg and P; and decrease the harmful effects of toxic heavy metals such as Al, Fe and Cd^39–42^. In addition, the positive Pearson’s correlation between Si-foliar concentration and Si shoot and root accumulation with the productive components showed that silicate application, in part, benefited wheat development.

The use of Si had little effect on NUE and grain yield associated with low and average N rates (0 to 150 kg N ha^−1^). In contrast, Detmann *et al*.^81^ and Neu *et al*.^25^ verified an increase of 2.3 and 11.5% in NUE, respectively, when Si was applied. Silicon levels in Rhodic Hapludox soils in tropical areas can be less than 1% due to the presence of extremely active desilification processes^82^. Additionally, some crops (for example, sugarcane, rice, wheat, and maize) can remove large amounts of Si from soil^43^, which can significantly reduce cereal yields if not properly restored^83,84^; thus, studies on Si application to improve NUE and N uptake should be performed. Although Si levels in soils vary, on average, between 2.8 and 16.8 mg dm^−3^ ^85^, increased grain yield is not always verified when available Si in soil is above 10.0 mg kg^−1 2,^ ^86,87^. The available soil Si contents in this study were close to this range (9.4 mg kg^−1^ at 0–0.20 m and 10.2 at 0.20–0.40 m, Table 1). In addition, more Si became available as a straw decomposed during the growing season (10.3 kg Si ha^−1^ and 37.6 of C/N ratio, Table 2). Some studies have reported that Si can be beneficial under stress conditions^88^. For example, Amin *et al*.^89^ observed that silicon application (Ca silicate dissolved in KOH at 71 °C) significantly increased plant height, stem diameter, number of leaves, cob length, number of grains, mass of 100 grains and maize grain yield under drought stress conditions. The fact that adequate amounts of Si were present in the soil used for this study and the lack of a positive response to added Si suggest that little to no biotic or abiotic stress was present during the growing seasons studied. Therefore, studies conducted under tropical conditions with Ca and Mg silicate application at soil amendment are needed to better understand the role of Si, applied alone or in combination with growth-promoting bacteria such as *A*. *brasilense*.

**Table 1:** Soil chemical attributes in 0–0.20 m and 0.20–0.40 m layers before the application of liming sources (before the beginning of field trial).

Soil chemical attributes	0–0.20 m layer	0.20 m–0.40 m layer
Total N	1.04 g kg^−1^	0.81 g kg^−1^
Si (CaCl_2_)	9.4 mg dm^−3^	10.2 mg dm^−3^
P (resin)	19 mg dm^−3^	17 mg dm^−3^
S (SO_4_)	10 mg dm^−3^	30 mg dm^−3^
Organic matter	21 g dm^−3^	16 g dm^−3^
pH (CaCl_2_)	5.0	4.8
K	2.1 mmol_c_ dm^−3^	1.2 mmol_c_ dm^−3^
Ca	19.0 mmol_c_ dm^−3^	11.0 mmol_c_ dm^−3^
Mg	13.0 mmol_c_ dm^−3^	8.0 mmol_c_ dm^−3^
H + Al	28.0 mmol_c_ dm^−3^	28.0 mmol_c_ dm^−3^
Al	1.0 mmol_c_ dm^−3^	2.0 mmol_c_ dm^−3^
B (hot water)	0.17 mg dm^−3^	0.11 mg dm^−3^
Cu (DTPA)	3.1 mg dm^−3^	2.1 mg dm^−3^
Fe (DTPA)	20.0 mg dm^−3^	10.0 mg dm^−3^
Mn (DTPA)	27.2 mg dm^−3^	10.7 mg dm^−3^
Zn (DTPA)	0.8 mg dm^−3^	0.2 mg dm^−3^
Cation exchange capacity (pH 7.0)	62.1 mmol_c_ dm^−3^	48.2 mmol_c_ dm^−3^
Base saturation (%)	55	42

n = 20.

**Table 2:** Nutrient accumulation in maize straw (2016 wheat predecessor crop).

N	P	K	Ca	Mg	S	Si
(kg ha^−1^)						
116	11.1	91	61	30	15.0	10.3
B	Cu	Fe	Mn	Zn	C/N ratio	
(g ha^−1^)						
136	266	2380	1132	239	38	

n = 10.

## Materials and Methods

### Field site description

**Figure 10:**
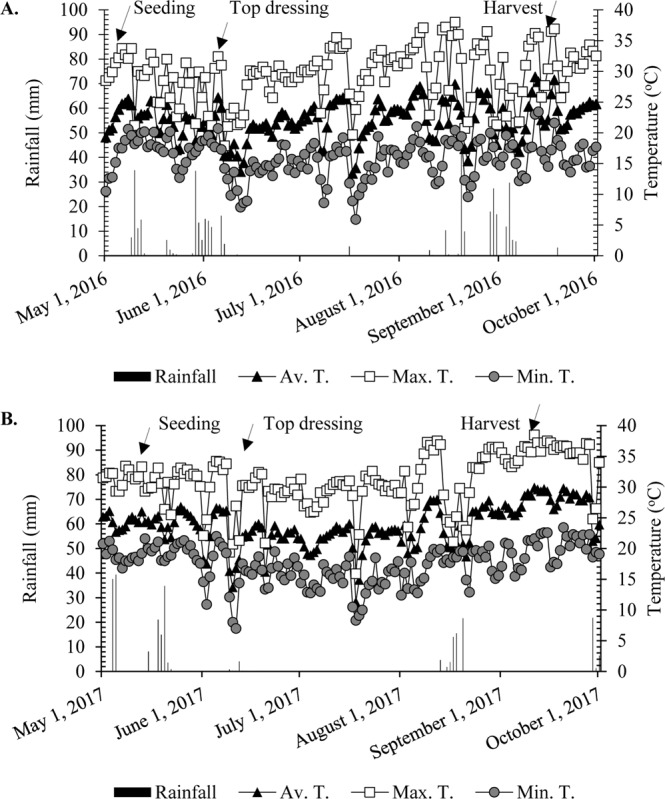
Rainfall, and temperatures (minimum, average and maximum) obtained from the weather station located in the Education and Research Farm of College of Engineering of Ilha Solteira/São Paulo State University (FEIS/UNESP) during the wheat cultivation in the period of May 2016 to October 2016 (**A**) May 2017 to October 2017 (**B**).

The study was conducted during the crop years of 2016 and 2017 under field conditions in Selvíria (Brazilian Cerrado – savanna region), state of Mato Grosso do Sul, Brazil (20°22′S and 51°22′W, 335 m above sea level (a.s.l.)) (Supplementary Fig. 1). The soil was classified as Clayey Oxisol (Rhodic Hapludox) according to the Soil Survey staff^90^. Soil chemical and physical properties were determined from soil samples collected prior to the lime application and analysed according to Raij *et al*.^91^. Total N was determined by the semimicro‐Kjeldahl method^92^. Si was determined after extraction in Ca chloride (0.01 mol L^−1^) according to Korndörfer *et al*.^93^ (Table 1). Particle size analysis was performed according to Embrapa^94^ and showed that the soil at the site had 471 g kg^−1^ of sand, 90 g kg^−1^ of silt and 439 g kg^−1^ of clay at the 0–0.20 m depth and 471 g kg^−1^ of sand, 82 g kg^−1^ of silt and 447 g kg^−1^ of clay at the 0.20–0.40 m depth. The experimental area was cultivated with annual grain crops for over 30 years. In addition, the area has been under no-tillage for the last 15 years. The last crop sequence prior to wheat (2016 and 2017 crop season) was maize (2015/16 and 2016/17 crop season). The temperature, rainfall, and air relative humidity observed during the study are presented in Fig. 10.

### Experimental design, trial establishment and management

The experimental design was a randomized complete block design with four replicates arranged in a 2 × 2 × 5 factorial scheme. There were two liming materials; Ca and Mg silicate, which was also the Si source (the composition was 10% of Si, 25% CaO, and 6% MgO) with an effective neutralizing power (ENP) of 88%, and dolomitic limestone (the composition was 28% CaO and 20% MgO) with an ENP of 80%. Two inoculations were used: with or without seed inoculation with *A*. *brasilense*. Five N rates were applied as side dresses (0, 50, 100, 150, and 200 kg ha^−1^). The experimental plots were 12 wheat rows of five meters spaced at a distance of 0.17 m, and the useful area of the plot was the central eight rows, excluding 0.5 m from each end.

Prior to the start of the study, lime was broadcast applied at the rate of 1.76 t ha^−1^ for the silicate and 1.94 t ha^−1^ for the limestone 30 days before planting maize (2015/16 predecessor crop) in one single application. No incorporation was performed as the area was under no-tillage. This approach is a common practice used by farmers growing wheat in this region of Brazil. The amount of lime applied was based on the initial soil analysis and the amount needed to increase the base saturation to 80%. At planting, the straw remaining from the previous crop was collected by removing the residue from 10 random points in the experimental area measuring 0.5 m^2^. The residue sampled was used for chemical tests to determine nutrient accumulation (Table 2). Additionally, a fertilizer application of 275 kg ha^−1^ of the granular fertilizer 08–28–16 (N-P_2_O_5_-K_2_O) was performed on the entire experimental site to supply phosphorus and potassium based on the soil analysis and wheat crop requirements^79^. During this nutrient application, 22 kg N ha^−1^ was applied to the entire experimental area. Therefore, the total amount of N applied in each treatment was the amount of N applied at the side dress (0 to 200 kg N ha^−1^ as indicated above) in addition to the application of 22 kg N ha^−1^. Although N side-dressing fertilization can be splitted in multiple applications, we have performed one single N application since the farmers growing cereals in the study area adopt this practice. Also, supplemental irrigation using a centre pivot sprinkling system was performed when needed at a water depth of 14 mm, which would minimize ammonia volatilization losses.

The *A*. *brasilense* strains Ab-V5 Ab-V6 (CNPSo 2083 and CNPSo 2084, respectively) were inoculated at a rate of 300 mL of liquid inoculant per 50-kg sack of seeds planted (guarantee of 2 × 10^8^ colony forming unity mL^−1^). These are commercial strains used in Brazil with the brand name AzoTotal^®^. These strains, when used under similar conditions (Brazilian savanna), have shown positive results in wheat development^1,2,24,70^. Seeds were inoculated after the seed treatment with insecticide and fungicide when the seeds were completely dry, one hour before planting. The insecticide used was fipronil (50 g of a.i. per 100 kg of seed), and the fungicides used were thiophanate-methyl + pyraclostrobin (45 g + 5 g of a.i. per 100 kg of seed).

Nitrogen treatments were applied manually to evenly distribute the fertilizer on the soil surface without incorporation. The amount of fertilizer needed per plot was applied between the wheat rows on June 8, 2016, and June 15, 2017 when the plants were in the vegetative stage equivalent to tillering - decimal growth stage GS21^95^. After the side-dress application, the experimental area was irrigated with 14 mm of water to minimize ammonia volatilization.

Before planting, weeds were controlled by the application of glyphosate (1800 g ha^–1^ of the active ingredient [a.i.]) and 2,4-D (670 g ha^–1^ of the a.i.). The wheat CD 1104 cultivar was mechanically sown on May 3 and May 10 for the 2016 and 2017 crops, respectively, at a planting density of 412 plants m^−2^. Wheat plants emerged five days after sowing, on May 8, 2016, and May 15, 2017,Supplemental irrigation using a centre pivot sprinkling system was performed when needed at a water depth of 14 mm. The herbicide metsulfuron-methyl (3 g ha^−1^ of a.i.) in combination with a vegetable oil adjuvant (720 g ha^−1^ of a.i.) were used for post-emergence weed control on May 28, 2016, and June 6, 2017, respectively. Harvest occurred on September 8, 2016, and September 12, 2017, which was 120 and 117 days after wheat emergence, respectively.

### Measurements collected

The leaf chlorophyll index (LCI) was measured in 10 plants per plot, using a portable non-destructive chlorophyll meter Falker ClorofiLOG® model CFL^−1^030^96^ during the flowering - decimal growth stage GS61^95^. Tissue N and Si concentrations were determined by collecting 30 flag leaves also during the flowering - decimal growth stage GS61^95^, from each plot^79^. In addition, the N and Si concentrations in the shoots and roots were also quantified, and N and Si accumulation values were calculated based on shoot and root dry matter. The nitrogen and Si analyses followed the methodologies proposed by Malavolta *et al*.^97^ and Silva^98^, respectively.

Shoot and root dry matter were measured during the female flowering stage in each experimental plot by collecting plants in 0.17 m^2^ (0.17 m × 1.0 m), and the values were extrapolated to kg ha^−1^. Plant height (m) was measured at maturity for 10 plants per plot. Height was measured from the ground surface to the apex of the spike. The number of spikes per meter was measured at maturity by counting spikes in 0.17 m^2^ (0.17 m × 1.0 m) per experimental plot. At the maturity stage, 10 wheat spikes also were collected in each experimental plot, following the methodology proposed by Galindo^1,2^ for the evaluations: (a) spike length (cm), distance from the apex to the base of the spike; (b) number of defective grains, by counting the number of undeveloped grains per spike; (c) number of spikelets, by counting all spikelets with grains; (d) number of grains per spikelet, by counting the number of grains in each spikelet; (e) number of grains per spikes, by counting the number of grains in each spike; (f) hectoliter mass, corresponding to the mass of wheat grains in a 100-L container determined on a ¼ scale after adjusting the water content of the grains to 13% (wet basis); and g) mass of 1000 grains (g), determined in precision scale 0.01 g, at 13% (wet basis); harvest index (HI), nitrogen use efficiency (NUE), calculated with the Eqs. 1 and 2 respectively, Moll *et al*.^99^ and grain yield determined by collecting the useful experimental plot area, adjusted to 13% (wet basis) and extrapolated to kg ha^−1^.

1 $${\rm{HI}},\,{\rm{expressed}}\,{\rm{as}} \% :[{\rm{Grain}}\,{\rm{Yield}}\div({\rm{Grain}}\,{\rm{Yield}}+{\rm{Shoot}}\,{\rm{Yield}})]\times 100$$

2 $${\rm{NUE}},\,{\rm{according}}\,{\rm{to}}:[({\rm{GYF}}-{\rm{GYW}})\div({\rm{amount}}\,{\rm{of}}\,{\rm{N}}\,{\rm{applied}})]$$

where GYF = grain yield with fertilizer and GYW = grain yield without fertilizer.

### Statistical analysis

Data were analysed by the Shapiro and Wilk^100^ test and Levene’s homoscedasticity test (*p* ≤ 0.05)^101^ and showed a normal distribution and variance. The data were analysed by ANOVA in a 3-way factorial design with liming sources, inoculation with *A*. *brasilense* and N rates considered fixed effects in the model using the ExpDes package. Mean separation was performed when significant factors or interactions were observed using Tukey’s test. Regression analysis was used to determine whether there was a linear or non-linear response to N rates using R software^102^. The heatmap was developed by calculating the Pearson´s correlation (*p* < 0.05) using the corrplot package to evaluate the relationship among the nutritional and productive components, NUE and grain yield parameters using R^102^.

## Supplementary information

Supplementary information.
