# Supplementary material for: Inoculation of Azospirillum brasilense associated with silicon as a liming source to improve nitrogen fertilization in wheat crops
Source: Sci Rep. 2020 Apr 9;10:6160. doi: 10.1038/s41598-020-63095-4 (PMC7145820; doi:10.1038/s41598-020-63095-4)
Supplement: Supplementary file 1 — Supplementary information. [file 41598_2020_63095_MOESM1_ESM.docx]

**Inoculation of *Azospirillum brasilense* associated with silicon as a liming source to improve nitrogen fertilization in wheat crops**

Fernando Shintate Galindo^1^, Salatiér Buzetti^1^, Willian Lima Rodrigues^1^, Eduardo Henrique Marcandalli Boleta^1^, Vinicius Martins Silva^1^, Renan Francisco Rimoldi Tavanti^1^, Guilherme Carlos Fernandes^1^, Antônio Leonardo Campos Biagini^1^, Poliana Aparecida Leonel Rosa^1^, Marcelo Carvalho Minhoto Teixeira Filho^1*^

^1^São Paulo State University (UNESP), College of Engineering of Ilha Solteira, Department of Plant Health, Rural Engineering, and Soils, P.O. BOX 15385-000, Av. Brasil Sul, 830 - Centro, Ilha Solteira, state of São Paulo, Brazil. *Correspondence should be addressed to M.C.M.T.F. (email: mcm.teixeira-filho@unesp.br)

Supplementary table 1. *F*-values for leaf chlorophyll index, N and Si-leaf concentration, N and Si-shoot and root accumulation, shoot and root dry matter, plant height, number of spikes per meter, spike length, number of defective grains per spike, spikelets per spike, grains per spikelet, grains per spike, hectoliter mass, mass of 1000 grains, harvest index, nitrogen use efficiency and wheat grain yield affected by N rates, liming sources, inoculation with *A. brasilense* and their interactions.

|  | Leaf chlorophyll index | | | | N-leaf concentration | | Si-leaf concentration | | N-shoot accumulation | |
| --- | --- | --- | --- | --- | --- | --- | --- | --- | --- | --- |
| *F*-values | 2016 | | 2017 | | 2016 | 2017 | 2016 | 2017 | 2016 | 2017 |
| N rates (R) | 6.936* | | 14.301** | | 23.220** | 26.364** | 1.785ns | 9.723** | 16.165** | 11.999** |
| Liming sources (L) | 2.743ns | | 2.127ns | | 2.825ns | 0.023ns | 15.884** | 11.525** | 1.688ns | 12.956** |
| Inoculation (I) | 5.362* | | 4.182* | | 0.361ns | 43.234** | 0.455ns | 73.769** | 9.958** | 2.425ns |
| R X L | 1.614ns | | 8.628** | | 0.208ns | 1.400ns | 1.522ns | 2.506ns | 2.047ns | 1.170ns |
| R X I | 0.678ns | | 1.592ns | | 7.996** | 5.001** | 0.061ns | 9.558** | 7.021** | 2.100ns |
| L X I | 0.592ns | | 0.062ns | | 25.401** | 0.188ns | 0.238ns | 0.001ns | 0.018ns | 0.397ns |
| R X L X I | 1.143ns | | 0.972ns | | 0.956ns | 1.792ns | 1.545ns | 2.643ns | 1.758ns | 2.127ns |
|  | Si-shoot accumulation | | | | N-root accumulation | | Si-root accumulation | | Shoot dry matter | |
| *F*-values | 2016 | | 2017 | | 2016 | 2017 | 2016 | 2017 | 2016 | 2017 |
| N rates (R) | 14.227** | | 12.711** | | 9.949** | 0.895ns | 6.920* | 2.741ns | 9.542** | 0.795ns |
| Liming sources (L) | 8.524** | | 42.567** | | 2.255ns | 0.839ns | 2.785ns | 60.105** | 16.221** | 18.687** |
| Inoculation (I) | 0.875ns | | 0.038ns | | 2.326ns | 2.740ns | 0.010ns | 2.723ns | 5.646* | 0.329ns |
| R X L | 1.484ns | | 2.088ns | | 1.009ns | 0.720ns | 0.122ns | 2.155ns | 1.683ns | 2.223ns |
| R X I | 1.075ns | | 1.327ns | | 0.078ns | 1.746ns | 1.208ns | 0.845ns | 0.477ns | 1.355ns |
| L X I | 1.631ns | | 2.230ns | | 0.058ns | 0.034ns | 0.476ns | 0.011ns | 0.008ns | 0.852ns |
| R X L X I | 1.042ns | | 0.677ns | | 0.512ns | 1.275ns | 1.241ns | 0.589ns | 1.915ns | 1.891ns |
|  | Root dry matter | | | | Plant height | | Spikes per meter | | Spike lenght | |
| *F*-values | 2016 | | 2017 | | 2016 | 2017 | 2016 | 2017 | 2016 | 2017 |
| N rates (R) | 5.209* | | 1.129ns | | 0.486ns | 1.269ns | 1.034ns | 10.834** | 7.780** | 18.154** |
| Liming sources (L) | 18.506** | | 14.451** | | 1.159ns | 1.980ns | 0.373ns | 2.113ns | 1.693ns | 0.559ns |
| Inoculation (I) | 2.268ns | | 2.226ns | | 2.457ns | 0.018ns | 0.166ns | 4.111* | 0.390ns | 0.001ns |
| R X L | 1.753ns | | 0.612ns | | 1.029ns | 0.545ns | 0.576ns | 2.183ns | 0.594ns | 1.014ns |
| R X I | 0.562ns | | 0.640ns | | 0.765ns | 0.954ns | 0.021ns | 2.287ns | 0.695ns | 1.040ns |
| L X I | 0.001ns | | 0.147ns | | 0.385ns | 0.480ns | 0.201ns | 0.473ns | 2.145ns | 0.706ns |
| R X L X I | 1.810ns | | 1.027ns | | 1.609ns | 0.058ns | 1.025ns | 1.918ns | 1.043ns | 1.028ns |
|  | Defective grains | | Spikelets per spike | | Grains per spikelets | | Grains per spike | | Hectoliter mass | |
| *F*-values | 2016 | 2017 | 2016 | 2017 | 2016 | 2017 | 2016 | 2017 | 2016 | 2017 |
| N rates (R) | 2.183ns | 1.346ns | 0.319ns | 6.898** | 1.554ns | 0.433ns | 1.235ns | 4.021** | 1.775ns | 2.124ns |
| Liming sources (L) | 1.720ns | 0.002ns | 5.496* | 0.044ns | 2.412ns | 2.243ns | 1.226ns | 2.172ns | 0.078ns | 0.005ns |
| Inoculation (I) | 9.706** | 2.056ns | 0.072ns | 1.959ns | 5.384* | 0.376ns | 2.125ns | 0.146ns | 0.007ns | 1.422ns |
| R X L | 0.369ns | 0.747ns | 1.144ns | 1.568ns | 1.062ns | 0.993ns | 2.257ns | 0.417ns | 0.948ns | 1.158ns |
| R X I | 2.275ns | 0.934ns | 0.324ns | 1.515ns | 0.915ns | 0.485ns | 1.344ns | 2.247ns | 0.923ns | 0.771ns |
| L X I | 0.134ns | 0.189ns | 1.149ns | 0.705ns | 0.440ns | 1.099ns | 0.021ns | 0.431ns | 1.954ns | 0.512ns |
| R X L X I | 0.428ns | 1.330ns | 1.177ns | 1.265ns | 1.612ns | 0.212ns | 2.052ns | 1.424ns | 1.032ns | 2.181ns |
|  | Mass of 1000 grains | | | | Harvest index | | Nitrogen use efficiency | | Grain Yield | |
| *F*-values | 2016 | | 2017 | | 2016 | 2017 | 2016 | 2017 | 2016 | 2017 |
| N rates (R) | 0.641ns | | 1.412ns | | 1.048ns | 0.126ns | 29.878** | 26.277** | 5.154* | 13.547** |
| Liming sources (L) | 2.237ns | | 1.607ns | | 4.573* | 0.176ns | 0.033ns | 1.491ns | 0.117ns | 0.418ns |
| Inoculation (I) | 5.757* | | 13.584** | | 0.168ns | 2.313ns | 1.211ns | 28.303** | 4.379* | 6.051* |
| R X L | 1.093ns | | 1.161ns | | 0.327ns | 0.704ns | 0.397ns | 0.810ns | 2.256ns | 3.932** |
| R X I | 1.478ns | | 1.171ns | | 0.206ns | 0.387ns | 0.551ns | 1.791ns | 4.256* | 5.588** |
| L X I | 0.540ns | | 6.162* | | 0.016ns | 1.081ns | 0.615ns | 2.272ns | 0.131ns | 0.086ns |
| R X L X I | 1.045ns | | 0.805ns | | 0.479ns | 0.089ns | 0.432ns | 0.858ns | 0.690ns | 0.297ns |

**, * and ns: significant at *p*<0.01, 0.01<*p*<0.05, and not significant, respectively.


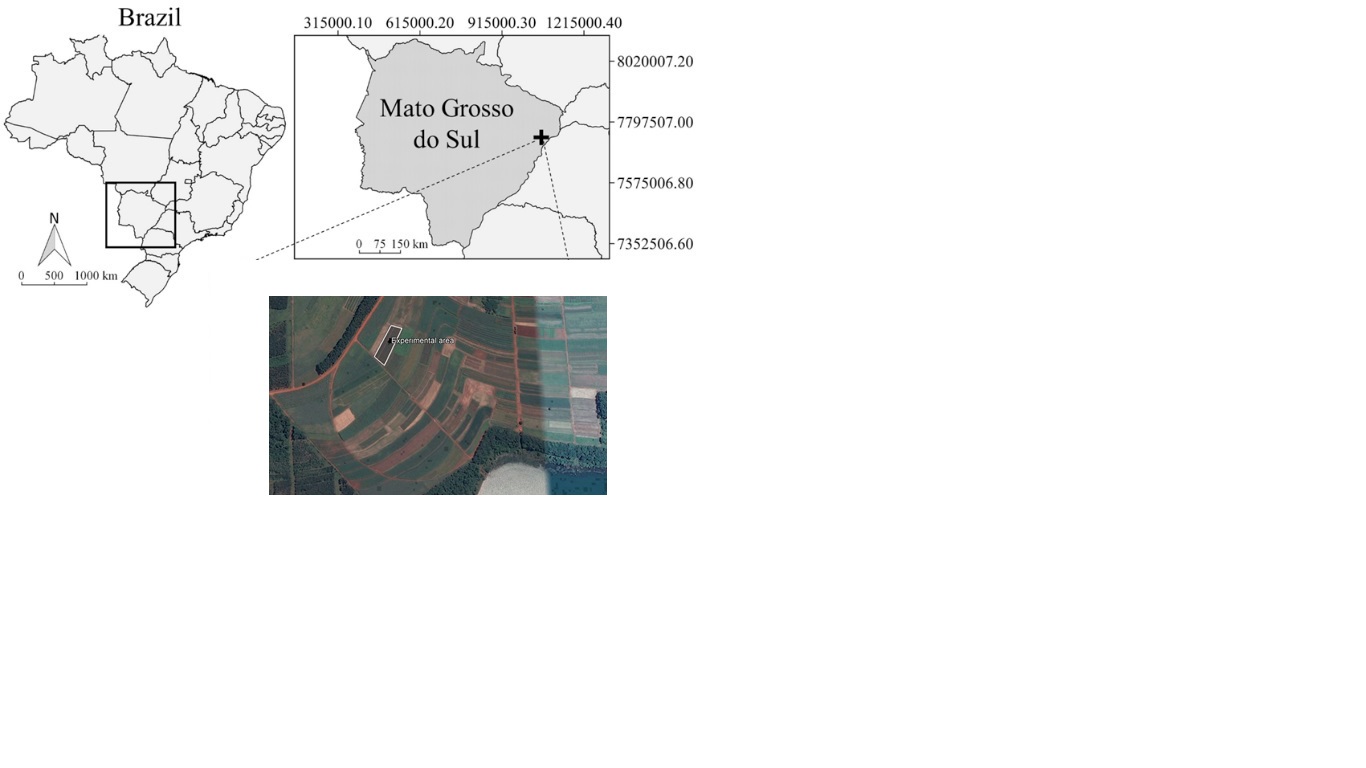


Supplementary fig. 1. Study area at Selvíria, state of Mato Grosso do Sul, Brazil (20^o^22′S, 51^o^22′W, the altitude of 335 m). Map created by using QGIS software and Google Earth program. QGIS Development Team (2019). QGIS Geographic Information System. Open Source Geospatial Foundation Project. http://qgis.osgeo.org. Projection System WGS 84 / UTM 21S [EPSG:32721]. Experimental area image obtained in Google Earth program, Google company (2019). Map data: Google, Maxar Technologies.
